# Massive Open Online Courses and intercultural competence: analysis of courses fostering soft skills through language learning

**DOI:** 10.3389/fpsyg.2023.1219478

**Published:** 2023-08-04

**Authors:** Laxmisha Rai, Chunrao Deng, Shuyang Lin, Liu Fan

**Affiliations:** ^1^College of Electronic and Information Engineering, Shandong University of Science and Technology, Qingdao, China; ^2^College of Professional and Continuing Education, The Hong Kong Polytechnic University, Kowloon, Hong Kong SAR, China; ^3^Management College, Ocean University of China, Qingdao, China

**Keywords:** intercultural competence, MOOC, culture, language education, soft skill, intercultural communication, education and technology, globalization

## Abstract

This paper aims to survey language teaching MOOCs that promote intercultural competence (IC). Specifically, the survey aims to identify the keywords most relevant to MOOCs with IC components, the languages taught, the offering countries, and the embedded soft skills. The selection of keywords is important because they can indicate connection between different disciplines. After trialing a broader set of keywords in several rounds of initial search, we identified five keywords that are the most relevant to language education with IC components on MOOCs: culture, intercultural, cross culture, multi culture, and society. Then courses with these five keywords on language learning are selected for further analysis. The results are summarized as follows: (1) Most language teaching MOOCs are found under the keyword “culture,” indicating a strong connection between language education and intercultural communication; (2) In terms of the target languages and the offering countries, it is found that English, Chinese, and Spanish are the major languages widely taught in the context of intercultural competence; China, United States, and Ireland are the three countries which offer the highest numbers of MOOCs in this respect; United States, United Kingdom, and Australia are the three countries which offer the highest numbers of MOOCs of different languages. The results indicate that a limited number of languages and offering countries dominate the language learning MOOCs. The study calls for a plurality of languages and cultures to be taught through MOOCs, making more diversified knowledge systems accessible to global audiences. (3) The language teaching MOOCs not only focus on language but also aim to foster five types of soft skills (language learning skills, communication skills, business and entrepreneurship skills, career development skills, and cultural development skills), suggesting that intercultural competence and its related soft skills are usually important components embedded in such MOOCs courses.

## 1. Introduction

In the recent decades, studies related to intercultural communication, intercultural competence, and intercultural communicative competence have become widespread especially due to the rapid development in communication through Internet and online platforms. Today, it is easier to interact with others, understand a foreign culture, or learn a new language, even though the parties from linguistically diverse backgrounds are culturally, behaviorally and ethnically different. A typical workplace in this globalized world is highly diversified. It is essential to understand and thrive in such environments for successful leadership. Terms such as inclusiveness, multi-cultural, intercultural, and global society are very common within multi-national companies, and supporting positive discourse on these topics is crucial for the development of an organization. This kind of discourse not only enhances effective communication within an organization, but also helps to foster greater collaboration between global corporations.

Recently, researchers have proposed several theories related to intercultural communication, cultural intelligence (Presbitero and Attar, 2018), intercultural competence (Byram, 2018; UNESCO, 2023; Zhang and Zhou, 2023), and intercultural communication competence (Byram, 1997; Collier, 2015; Koester and Lustig, 2015). Although these terms are used slightly differently in the literature, they are interchangeable in general. To define intercultural communication, it is important to define culture. According to Terpstra’s (1987) definition, culture is the integrated sum of learned behavioral traits that are shared by members of a society. Intercultural communication refers to the communication between people from two different cultures. Intercultural competence is defined as “a complex of abilities that are needed to interact with people from other cultures adequately and effectively” (Wolff and Borzikowsky, 2018, p. 1). The main goal is to reduce the differences between individuals’ cultures, beliefs, and experience; and develop abilities to interact with people from diverse backgrounds, thus reducing the cultural barriers. Similarly, the term “Intercultural Communication Competence” (ICC) has also been widely used. Hollingsworth et al. (2021, p. 154) pointed out that “ICC is the ability to communicate effectively and appropriately in various cultural contexts and some of the key components include motivation, self-and other knowledge, and tolerance for uncertainty.” While ICC focuses on specific issues such as motivation, self, and knowledge, Wolff and Borzikowsky (2018) move further and develop a model, where interacting with people from different cultures is of prime importance.

One main purpose of intercultural research is to understand intercultural competence, skills one needs to understand different cultures, backgrounds, beliefs, and experiences effectively while working in a global organization. Some researchers have listed features of intercultural competence (IC). According to Monash Intercultural Lab (2023), the main components of IC include skills, attitudes, culture and communication. The skills include listening to others, analyzing, observing, and thinking critically. Behaviors such as openness, curiosity, and respecting others are part of the attitude components. The culture aspects include one’s identity, belief, and values. The communication components include language skills, non-verbal behavior, and making dialogues. It is believed that having strong IC skills is essential in this globalized world for individuals to increase their soft-skills and to become an attractive asset while working in a global organization.

In recent years, it is common to see the trends of migration and change of citizenship. Many migrate to other countries to obtain better employment, higher studies, or better healthcare. In these circumstances, learning a foreign language and adjusting to a new culture are essential requirements. In addition to the developments in globalization, in many countries learning a foreign language such as English is an unavoidable trend to enhance higher communication competence among learners in the age of glocalization (combination of globalization and localization; Rai and Deng, 2016a). The rise of the Internet has enabled language courses to be delivered to learners with different cultural backgrounds in geographically distant locations. Considering the significance of IC in higher education, several researchers have studied the importance of IC in education, online education, and foreign language learning. Massive open online courses (MOOCs hereafter) have gained wide attention and reputation for online education in recent years.

Since the first offering of a Massive Open Online Course (MOOC), the body of literature on MOOCs has examined various aspects of MOOCs, including learner behaviors (Kizilcec et al., 2013; Rai and Deng, 2016b), enrollment and completion (Jordan, 2014), assessment (Xiao et al., 2019), and learner motivations and intentions (Moore et al., 2021). Some researchers used keywords to search and review journal articles to reveal the major themes and concepts covered in the publications (Zawacki-Richter et al., 2018). However, there is a lack of research that uses keywords to collect and analyze empirical data on teaching topics related to IC provided in MOOC platforms. Keyword research is significant because the use of keywords implies the connection between disciplines. When relationships among the keywords are drawn as a keyword network, network metrics can be used to elicit novel insights and the evolution of knowledge (Choi et al., 2011). In addition, while MOOCs are claimed to disseminate knowledge globally, they have also been problematized as a new form of coloniality because of their dissemination of Western-centric epistemologies (Altbach, 2014; Adam, 2019). Therefore, an important but under-researched topic is which countries offer MOOCs that promote IC or ICC and which cultures and languages are taught.

This study aims to identify the keywords most relevant to MOOCs with IC components, the languages taught, the offering countries, and the embedded soft skills. Below are the three specific aims:

- To identify the keywords that indicate connection between cultural components and MOOC language courses.
- To identify the type of languages and host countries in MOOC courses with IC components.
- To analyze the types of soft skills promoted by MOOC language courses.

By identifying the keywords, the study will examine what cultural elements are connected with language education. By revealing the type of languages and offering countries, the study will show the epistemic underpinnings of such MOOC courses. If epistemic injustices are identified in MOOC language courses that promote ICC, the results will point to the need of acknowledging and legitimizing diversified knowledge systems.

This paper is organized as follows: Section 2 provides the background and related work. Section 3 presents the research methodology. Section 4 describes the details of results and discussion. Section 5 provides the concluding remarks.

## 2. Literature review

### 2.1. Intercultural competence and education

In earlier studies, researchers have considered the role of IC in the business domain, multinational companies, and international experience. Presbitero and Attar (2018) assessed employees working in multinational companies within intercultural contexts in Australia. Leung et al. (2014) evaluated the current measurement of intercultural competences and provided deep insight into distinguishing features, attitudes, and capabilities which predict success in intercultural contexts. Johnson et al. (2006) discussed the model of cross-cultural competence in the international business domain. Wolff and Borzikowsky (2018) examined development of intercultural competence during international experiences and argued that “there is a growing interest to foster intercultural competence development by educational stays abroad” (p. 1).

Considering students in culturally diverse schools and their intercultural competence, Schwarzenthal et al. (2020, p. 341) showed that “contact and cooperation, color-evasion (as in emphasizing a common humanity), and multiculturalism in the classroom are uniquely related to aspects of students’ intercultural competence.” Ferreira-Lopes et al. (2021) studied business students’ intercultural competence through intercultural virtual collaboration and showed that their results “fostered a positive attitude towards intercultural relationships, increased students’ cultural knowledge and awareness and equipped students with skills to work in diverse teams” (p. 338). Liang and Schartner (2022) tried to address whether culturally mixed group work could contribute to students’ intercultural competence development. They found that cultural mixed group work could increase their cultural awareness and foster ways to develop intercultural competence.

Several studies have focused on intercultural competence among students (Müller et al., 2020), as well as intercultural communicative competence in online teaching (Shen, 2021). Zhao et al. (2018) discussed intercultural competence in the context of higher education focusing on university educators. Similarly, there are other studies on intercultural competence and its significance on learners of English as a foreign language (EFL), distance learners, and foreign language learners. Huang (2021) proposed the development of intercultural communicative competence in foreign language classrooms and argued that providing explicit instruction facilitates the development of ICC among EFL learners. Sierra-Huedo and Nevado-Llopis (2022) showed the need for more intercultural learning interventions to develop intercultural competence in higher education to educate students as global citizens. Similarly, the development of students’ intercultural competence and their intercultural experience were studied in Lantz-Deaton (2017). This study was conducted with a cohort of first year United Kingdom and non-United Kingdom students studying in an internationally diverse campus. The development of students’ intercultural competence in FLT contexts was studied (Bouslama and Benaissi, 2018). Its goal was to evaluate how Algerian EFL teachers perceived the concepts of culture and intercultural competence in ELT contexts.

Imanova (2021) studied the advantages and disadvantages of promoting intercultural competence in foreign language learning. This study specifically focused on learning a foreign language through distance education within the context of higher education in Azerbaijan. The author highlighted that there was not much difference in opportunities to practice ICC by using modern technologies such as online learning as compared to face-to-face learning. Mu et al. (2022) studied how individual experience plays a role in development of students’ intercultural competence while studying abroad, and how such experience positively or negatively impacts their intercultural development. The purpose was to find the reasons why to some students, studying abroad brought positive changes in intercultural competence, while to others, it was not the case.

Lloyd and Härtel (2010) developed an intercultural competencies classification system and examined the role that IC played in facilitating positive individual outcomes. Hang and Zhang (2023) reviewed studies published between 2010 and 2021 to analyze the intercultural competence developmental processes of university and college students. They presented a systematic review to analyze empirical studies on university and college students’ IC developmental process based on reasoning approach, research design, influential factor, and outcome. Zhang and Han (2020) studied the effectiveness of technology-mediated cross-campus teaching and learning on students’ intercultural competence. Mu and Yu (2023) studied how the intercultural competence of college business English undergraduate students in China was cultivated by including intercultural perspectives in the classroom and by integrating intercultural language teaching.

### 2.2. Technology and intercultural competence

Sherwood et al. (2018) presented how technology such as online learning and Skype-based learning play a role in intercultural competence development among students. Shadiev and Sintawati (2020) studied how technology supports intercultural learning and education by reviewing 25 articles published between 2014 and 2019. This study found that videoconferencing and email were the most frequently used technologies; English was the most frequently used language; and United States and China were the most frequently involved countries.

Dhar and Yammiyavar (2012) selected the publications to evaluate the role of culture in the design of cross-cultural educational products and to find how they influence the acceptance and usability of a globalized online learning environment. Shadiev et al. (2015) studied cross-cultural understanding and collaborative learning implementation in an online environment. In addition, Shadiev and Sintawati (2020) studied intercultural learning activities supported by VR technology and found that such learning helped facilitate intercultural competence development. Litvinova et al. (2021) investigated intercultural communicative competence through online learning where undergraduate students of medical and linguistic specialties from state universities were involved. They found that the linguistic students were more aware of intercultural competence than the medical students. Hackett et al. (2023) investigated the effectiveness of collaborative online international learning (COIL) on intercultural competence development in higher education by gathering both quantitative and qualitative data. They found that COIL helps to develop intercultural competence in terms of cultural intelligence, yet such increase was not observed in students who had already been exposed to international experiences. Considering the technology-assisted cross-cultural communication and communication competences, Guo and Hwang (2022) studied the foreign language and cultural competence. They found that virtual exchange intercultural communication project can contribute to the development of university students’ intercultural competence and online communication.

### 2.3. Massive open online courses and cultural inclusiveness

In the past decade, massive open online courses (MOOCs) have gained worldwide attention, especially after MIT and Harvard University jointly announced offering courses for free through edX for the first time in 2012. Since then, many universities and organizations have joined as partners, and high-level courses are provided to online learners. Simultaneously, apart from edX, many MOOC providers also started offering courses for free, such as Coursera, Udacity, FutureLearn, iVersity, etc. At first, most of the courses were offered for free, but now learners are sometimes required to pay to utilize the full benefits of MOOCs. MOOCs have revolutionized online education and reached almost every corner of the world. Even though MOOCs has the power to influence learners around the world, the success rates of courses are very low, sometimes as low as below 1%. Rai and Deng (2016b) identified the factors that influence success and failure rates in MOOCs. Despite the high failure rates, learners still find MOOCS valuable and believe there are several ways they can be successful in learning through MOOCs (Rai, 2019).

Considering the development of MOOCs, several recent studies have described how MOOCs can influence cultural aspects and what role MOOCs can play in minority education. Li et al. (2016) developed an adaptive MOOCs platform to assist teachers and students in developing various versions of ethnic cultures. Considering MOOCs for digital humanities, Kappas et al. (2017) suggested a way to create MOOCs for Greek Arts by making use of digitized cultural resources to train secondary education teachers to use University Open Courses. Duangjinda et al. (2021) developed a multicultural society in MOOC to enhance the cultural awareness of undergraduate students and found that multicultural awareness was significantly increased. In Taheri et al. (2019), a design-based approach to evaluate the culturally inclusive MOOCs was conducted. The authors argued that rich learning experience in MOOCs can be gained by having diversity within MOOCs.

### 2.4. Massive open online courses as digital neocolonialism

While MOOCs have been positively viewed by some as making education more accessible, it has been problematized as a form of digital neocolonialism and education hegemony (Altbach, 2014; Adam, 2019). Studies that investigate trends of MOOCs show that the developers of MOOC platforms, the designers of MOOCs, and the teachers which deliver the MOOCs are mostly from the Western culture. The major MOOC platforms, such as Coursera, edX, Udacity, and Futurelearn, mostly originate from top universities in the United States and the United Kingdom and Silicon Valley corporations (Shah, 2018; Adam, 2019). Most MOOCs offered on Coursera and edX were from Western countries including the United States, the United Kingdom, Canada, and Australia (Altbach, 2014). Only 7.3% of the MOOCs on Coursera were from the Global South countries (Adam, 2017). There was no MOOC platform from Africa and very few MOOCs from African institutions (Adam, 2019). In other words, these studies show that Western epistemologies dominate the entire cycle of MOOCs production, and this has important implications for the developing countries.

Under the dominance of Western epistemologies, it may be difficult for the local knowledges to gain acknowledgement and spread its influence. For example, Adam’s (2019) keyword search of MOOCs with ‘Africa’ in the title shows that MOOCs that cover topics about Africa were mostly hosted by Western institutions and the content was not adapted to the local communities. In this case, both the offering institutions and learners were from outside of Africa. This is similar to what Said (1978) observed about the Western invention of the Orient—local knowledge does not own its narrative but is represented through Western’s lens. Although MOOCs platforms seem to distribute knowledge globally, with the technology and resources dominated by the Western countries, they are creating a digital divide that may reinforce the Western-centric epistemologies as normative ones and hinder the marginalized and indigenous ones (Altbach, 2014; Adam, 2019). While Altbach (2014) does not deny the merits of the current Western-centric academic culture, he calls for a plurality of scientific inquiry and methodologies in academic discussions. He notes that the Western academic system exerts more influence on social science and humanities subjects than sciences ones in terms of “the academic traditions, methodological orientations, and teaching philosophies” (p. 4).

Given the heavier influence of Western epistemologies on social sciences and humanities subjects, it is particularly important to examine which countries offer MOOCs that promote intercultural competence, and what cultures are focused. According to Byram (1997), intercultural communicative competence (ICC) involves knowledge, attitude and skills needed for a learner to interact effectively with people from different cultures. One essential component of ICC is critical cultural awareness (CCA), which is defined as “(a)n ability to evaluate critically and on the basis of explicit criteria perspectives, practices and products in one’s own and other cultures and countries” (p. 53). Given the epistemic injustices on MOOCs identified in the previous studies, we identify the need to examine the landscape of MOOCs that promote intercultural competence, a topic that has received scarce attention. If the knowledge, attitude, and skills embedded in the MOOCs are underpinned by the dominant western-centric epistemologies, one may reasonably doubt whether the learners can develop CCA that enables them to conduct higher-order thinking about different cultures and traditions, e.g., taking different perspectives, analyzing the construction of knowledge, and questioning what is presented as normative.

### 2.5. Language learning and soft skill development

Hard skills and soft skills are both essential for an individual working in a global organization. Employers use various measures to select the job applicants based on their hard and soft skills (Wings et al., 2021). Hard skills matter while acquiring employment, whereas soft skills ascertain prospects of employability and enhances one’s interactions, job performance, and career growth (Vasanthakumari, 2019). Soft skills such as e-skills, critical thinking, and research skills are not only necessary for educators, teachers, and students, but also play an important role for students to access the labor market (Agrusti et al., 2017). According to Medvedeva et al. (2022), soft skills are of critical importance especially in developing one’s academic and professional achievement. Soft skills are also highly relevant in continuously changing environment and especially play a significant role in enhancing graduate employability (Succi and Canovi, 2020).

Soft skills are defined as “the attributes and behaviors that describe how a person approaches their tasks” (Coursera, 2022). Hard skills generally refer to academic skills, technical skills and subject knowledge, which are mensurable skills, for example, skills such as computer programming, writing, and data analysis. Banupriya (2011) referred to soft skills as “cluster of personality traits, social graces, facility with language, personal habits, friendliness, and optimism that marks each of us to varying degrees” (p. 109). Kopolovich (2020) defined soft skills as “non-technical skills, enable individuals to interact effectively and cooperatively with others” (p. 92). Dash et al. (2020) showed that soft skills are related to language learning, concepts of behavioral aspects, and grooming etiquette for better employability.

Medvedeva et al. (2022) proposed interactive methods of assessing students’ soft skills within the context of a foreign language course. They have focused on monitoring the progress of soft skills such as problem solving, teamwork, leadership, time management, technology skills, analytical, and creative thinking. Andrievskikh and Lapina (2021) suggested ways of integrating soft skills into university English language classes. Dash et al. (2020) described the importance of better communication in English and how this contribute to soft skill development especially while graduates are seeking employment. Julián and Ramírez (2021) discussed the importance of soft skills such as communication, problem-solving, teamwork, time-management or leadership, and how they influence the profile of engineering students. They have shown how soft skills and English proficiency can be enhanced while studying through project-based learning and in flipped classrooms.

Korolyova et al. (2021) discussed how learning a foreign language contributes to the development of soft skills among engineering students and ways of cultivating soft skills. They have described how different soft skills can be implemented in foreign language training, including communication skills, teamwork skills, presentation skills, negotiation skills, leadership skills, and intercultural skills. Kopolovich (2020) proposed the development of a soft skill MOOC with the title “Negotiation Management” for teaching soft skills and stressed the importance of teaching soft skills via MOOCs. Cinque (2017) showed how MOOCs can help in training 21st century skills including both soft skills and digital soft skills. They have analyzed 151 MOOCs on soft skills and qualitatively compared four creativity MOOCs. As the majority of the MOOCs are in English, they have highlighted how this creates a language barrier to participation in MOOC learning. Zueva (2019) described different ways of soft skill development in foreign language training and the importance of soft skills connected with communicative skills in professional development.

García-Martína and García-Sánchez (2020) studied how MOOCs play a role in teaching various personal skills and efficacy of instructional approaches in a MOOC. They argued that personal skills can be acquired by efficient instructional approaches. Hamori (2018) views that companies can use MOOCs to develop core competencies of their employees. She further argued that companies have opportunities to utilize MOOCs to improve their professional skills and improve the career prospects of employees by filling the knowledge gaps. Similarly, Calonge and Shah (2016) pointed out that MOOCs can facilitate in reducing the gap in skills that graduates have before employment and skills needed by the employers. Edelsbrunner et al. (2022) showed that MOOCs can reduce digital skill gaps and can be a great resource in developing digital skills among employees. Manning et al. (2014) studied how MOOCs have the inherent capacity to build knowledge and skills among language learners.

Our literature review shows that while many studies (Sherwood et al., 2018 for example) have examined how technology plays a role in intercultural competence development among students in long distant learning such as Skype-based learning, very limited studies have directly related IC to massive open online courses (MOOCs) or investigate how learners are expected to improve their IC skills through MOOCs. The study of how MOOCs support the teaching and learning in IC is significant for two main reasons. First, there is a significant difference between MOOCs and traditional online lessons, such as Skype lessons. Most online lessons allow a certain level of synchronous interaction, while many MOOCs mainly provide one-way lecturing courses supported with assessment. Secondly, MOOC providers as one of the biggest and most reputed online learning platforms have gained worldwide attention since edX platform provided the first free course in 2012.

In addition to how MOOCs as a reputed e-platform can support IC teaching, this study also aims to fill the gaps in the existing research. For example, most of the current IC and ICC studies have focused on traditional classrooms supported by technology, where students and teachers have synchronous interaction. How the non-synchronous e-platform can achieve the goal needs to be explored. Also, when teaching foreign languages and cultures, it is important to cultivate critical cultural awareness (Byram, 1997), enabling learners to exercise critical thinking when encountering different cultural traditions. Previous studies have identified the digital coloniality of MOOCs through disseminating Western-centric epistemologies (Altbach, 2014; Adam, 2019). However, little is known as to the epistemic underpinnings of MOOC language courses that promote ICC. Analyzing the languages taught and offering countries involved in the MOOCs will provide insights to this topic. In addition, some studies have suggested ways of integrating soft skills into university English language class (e.g., Medvedeva et al., 2022). But there is a lack of research that bridges the two areas to explore how soft skills can be embedded in the language learning courses with IC components. Considering these, this study tries to answer three research questions (RQs).

- RQ1: Which culture-related keywords are embedded in the titles and introductions of language education MOOCs that promote intercultural competence?
- RQ2: Which languages and offering countries are involved in the MOOCs that promote intercultural competence?
- RQ3: What soft skills are embedded in the language learning courses with IC components in MOOCs?

## 3. Methodology

**Figure 1:**
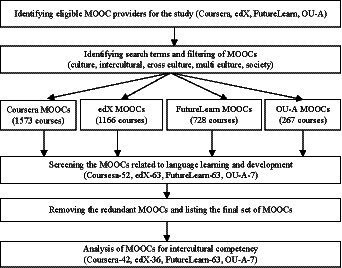
Research methodology.

This study employs a research methodology adapted from Shadiev and Sintawati (2020) to search, screen, and select data systematically. Our literature review shows that this methodology is well-accepted in intercultural research (Çiftçi, 2016; Avgousti, 2018). As shown in Figure 1, four main stages were involved to collect and analyze data. The sections below start from reporting how the platforms, subject areas and keywords were identified. We then describe how the data were screened. After that, we provide the data analysis method, informing readers what categories were used and how the data answer our research questions. Finally, reliability measures are discussed.

### 3.1. Identifying MOOC platforms

A website (Social Learning, 2023) lists the top 10 popular MOOC providers in recent years. They are Coursera,^1^ edX,^2^ FutureLearn,^3^ Udacity,^4^ NovaEd,^5^ iVersity,^6^ Canvas,^7^ Open Universities—Australia,^8^ Openlearning,^9^ and Udemy.^10^ It is evident from online search results that these MOOC providers are indeed popular. However, using “culture” as the main keyword in the initial stage, we found that providers such as Udacity, iVersity, Canvas, Openlearning and NovoEd returned less than 20 search results. The reasons for such low numbers of results are because some of these providers currently focus on courses on different topics such as technology and computer science (as in Udacity and iVersity). Other platforms such as Alison^11^ and Udemy returned more results (note that Alison is not listed as a top 10 MOOC provider as described in Social Learning, 2023), but they are not considered for the survey for the following reasons: Firstly, the majority of courses were developed by these providers themselves. For example, in Udemy, an instructor can build a course of his/her choice and offer it online. In most of the popular course providers such as Coursera, edX, FutureLearn, and Open Universities, Australia (OU-A), the courses are mainly offered by universities or organizations in contrast to Udemy and Alison, where vast numbers of courses are generated by the individuals or companies themselves. Moreover, in edX, some courses are also offered jointly by more than one organization. To keep uniformity in terms of course generators, Alison and Udemy are not considered. In summary, only four MOOC providers are considered for this survey: Coursera, edX, FutureLearn, and OU-A.

### 3.2. Identifying subject areas

In the initial stage, we reviewed literature and found that the major areas include language education (Byram, 2020), communication (Collier, 2015), sociology (Little et al., 2016), arts (Mahgoub, 2007), humanities (de Zepetnek, 2007) and international business (Schmidt et al., 2007). We used these keywords suggested by our literature review for initial search, including “intercultural,” “culture,” “multi culture,” “cross cultural,” “society,” “sociology,” “art,” “global,” “communication,” “connected,” “humanities” and “international.” This search resulted in huge numbers (5,762), clearly indicating a need for us to focus on one specific area so that we can develop the depth of the research. According to Byram (2020), language education could be one of the most suitable areas although it does not need to claim sole responsibility for the teaching and assessment of ICC. Language education, particularly foreign language education (FLT), is at the center of this concern as it requires learners to engage with both familiar and unfamiliar cultures through the medium of the target language. Furthermore, with the development of communicative teaching approach in FLT, a central aim of teaching is to enable learners to use the target language to interact with people from a different cultural background (Ellis, 1996). Initial search shows that language education MOOCs are highly related to the intercultural communication topics. Therefore, we decided to limit the scope of our study to this aspect.

### 3.3. Identifying keywords

Another important step is to screen and select keywords. As discussed earlier, in initial search, we used the most obvious keywords suggested by literature review, which, however, generated a huge number of results. Therefore, we screened these keywords with closer scrutiny. Among other keywords, “communication,” “global,” and “connected” were three important keywords we used in our initial search as communication is the main bridge between cultures in this connected world (Collier, 2015). However, these three keywords were removed from the searching list because quite a lot of results generated by “communication” “global” or “connected” were either not under language education category or were included by using other keywords. Although several courses were marginally related to language education, many topics were beyond our scope, for example the topic about intellectual property and information ethics. In contrast, language courses could be found more effectively by the keywords “cultural,” “intercultural,” “cross culture” and “multicultural” because the default keyword search in MOOCs is to search any field, which means it would search not only the course title, but also introduction and syllabus. As a result, under the keyword “cultural,” we found many language education courses with the word “communication” in the title, for example, *Take Your English Communication Skills to the Next Level* provided by The Georgia Institute of Technology in Coursera (See Appendix A, number 3) and *Japanese Pronunciation for Communication* offered by the Waseda University through edX (see Appendix B, number 16). In other words, keywords such as “culture,” “intercultural,” “cross culture,” and “multi culture” are sufficient and more effective because these words reflect the super set of keyword “communication” or “connected.” Our analysis also shows that most results generated by “sociology,” “art,” “international,” and “humanities” were not related to language education. For example, one of the courses generated by “international” in edX was *International Economics and Trade*, which was a business subject. As a result, these key words were removed from the list. After completing the above steps, we identified five key words: culture, intercultural, cross culture, multi culture, and society.

### 3.4. Screening of results

After we used the five keywords to search in the four MOOCs providers, we obtained 3,734 results. To make the search results more relevant, we include the courses (1) which have particular keywords in the title or course introduction; (2) which teach languages, such as Chinese, English, and Spanish; and (3) which are offered by universities, organizations, and institutions. After applying these three criteria, the results obtained included courses, short courses, ExpertTrack courses, subjects, certificate courses, topic discussions, career advice and titles of under-graduate and post-graduate degrees. To further narrow down the search results, we manually removed the results which did not meet the criteria of a course, for example, titles of degrees, certificates, topics, and discussions. Topic results that provide a list of subtopics rather than referring to any particular courses were also excluded. Some ExpertTrack courses which may not be of high relevance were eliminated. Similarly, career advice, subjects (not courses), and introductory information on a partner organization were also excluded.

After screening out all the results which did not meet the criteria of a course, the next step was to manually select IC courses that focus solely on language learning. Some courses had the keyword “culture,” but our manual examination showed that they did not include IC components, e.g., the course “*Human-Computer Interaction II: Cognition, Context & Culture*.” Several results which included the name of a language in the title, but not related to language education. For example, as the course “*Introduction to Modern Italian Literature and Culture*” does not focus on language learning or the usage of a language in communication settings, so they were also removed. We cross-checked the results for further verification.

To sum up, as shown in Figure 1, the first step was to identify the eligible MOOC providers. The second step was to decide the subject areas and keywords used for search by reviewing literature and conducting an initial search in MOOCs. After obtaining the complete set of results from four MOOCs providers, in the subsequent step, we only selected MOOCs related to language learning. This was done by verifying the titles, introduction, and syllabus of each MOOC. Finally, the obtained MOOCs were analyzed for intercultural competency features. After the final verification, we reduced the number of courses to 138 as shown in Figure 1.

### 3.5. Data analysis

We entered the obtained data through Excel sheet by category to generate graphs for analysis. Five categories were used to answer the research questions, including keywords, language types, host countries, number of languages involved in host countries, and the type of soft skills. The analysis of keywords allows us to understand the key cultural components in MOOC language courses. For example, the keyword “society” implies the connection of sociolinguistic elements in the course content, and “multi culture” indicates the inclusiveness and interaction of more than two languages in the course. The categories of host country and language types tell the readers what countries contribute to MOOCs and what types of languages gain attention by providers and audience. The comparison between the type of language and the host country also allows us to understand what type of culture attracts attention for what particular target audience. For example, we found a Japanese language course offered by TsinghuaX in China with Chinese as the medium of instruction, suggesting that the Japanese culture could be attractive to Chinese audience from provider’s perspective. The examination of the landscape of MOOCs helps us to explore whether Western-centric epistemologies dominate the ICC courses. Soft skill as a category was used because it was one main theme emerged from our initial analysis during the searching period. The analysis of soft skills is important because it shows that the goal of language MOOCs is more than merely developing language speaking, reading, writing or translation skills.

### 3.6. Reliability

To ensure reliability, the three authors discussed the searching and filtering method before the data collection started. We then used a combination of different keywords to conduct a trial searching practice separately. We kept close communication during the pilot searching period and discussed the discrepancies and resolved differences in meetings. After achieving over 90% agreement about our searching results, the first author used the agreed steps to collect data in the four MOOC platforms. After that, the results were cross checked by the second and third author, respectively, to ensure reliability.

## 4. Results and discussion

Results will be presented to address the research questions in turn, starting with keywords in language learning MOOCs (RQ1), the types of language and countries that are involved in the courses (RQ2), followed by the soft skills embedded in these language learning courses (RQ3).

• RQ1: Which culture-related keywords are embedded in the titles and introductions of language education MOOCs that promote intercultural competence (IC)?

Table 1 summarizes the list of search results generated from these four MOOC providers after applying the five key words (culture, intercultural, cross culture, multi culture, and society). There were 1,573, 1,166, 728, and 267 search results from Coursera, edX, FutureLearn, and OU-A, respectively, amounting to 3,734 search results. As mentioned in the Methodology section, we then further narrowed down the scope of our search by applying a set of criteria, and then obtained 185 results which were language education courses (October 18, 2022). We further removed redundant results from the 185 language learning courses. By using the keyword search, some courses appearing under more than one keyword were counted more than once. After excluding them, we obtained 138 MOOCs related to language learning. The complete details of the MOOCs are provided in Appendix A–D, which list titles and related details of the language learning related MOOCs offered by Coursera, edX, FutureLearn, and OU-A, respectively.

**Table 1:** Total number of MOOCs obtained by using five keywords and number of language learning MOOCs that meet our criteria.*

	MOOC providers				
Keywords	Coursera	edX	FutureLearn	OU-A	*Total*
culture	819 (42)	469 (34)	322 (50)	163 (5)	131
intercultural	36 (3)	241 (18)	19 (2)	3 (1)	24
cross culture	156 (7)	106 (4)	14 (0)	11 (0)	11
multi culture	22 (0)	10 (0)	9 (1)	2 (0)	1
society	540 (0)	340 (7)	364 (10)	88 (1)	18
*Total*	1,573 (52)	1,166 (63)	728 (63)	267 (7)	3,734 (185)

* The number in brackets indicates the number of language learning MOOCs that meet our criteria.

**Figure 2:**
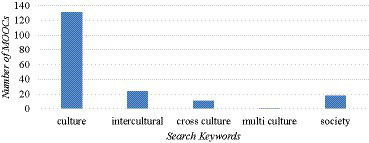
Number of Massive Open Online Courses (MOOCs) of language learning demonstrating intercultural competence based on five keyword searches.

Figure 2 shows the number of language learning courses obtained by each keyword search. It is found that most of the MOOCs are obtained under the keyword “culture,” accounting for more than 70% (131/185) of the total search results. This result is quite interesting because even though we have used other keywords such as “intercultural,” “cross culture,” “multi culture” and “society,” the number of courses obtained under “culture” keyword still significantly outnumbered the other search results, showing that language learning MOOCs predominantly emphasize aspects in one culture. This is understandable because when a language is taught, the aspects related to how the language is used in the specific culture naturally form the focus of the teaching. This finding confirms the view in literature that language and culture cannot exist without each other (Jiang, 2000; Mazari and Derraz, 2015). Culture plays a very significant role in foreign language education since words and phrases of that language refer to internal meanings of its culture, creating a reality and a semantic relationship that learners must understand. Some courses use “society” as the main keyword in the title and course introduction, indicating that the sociolinguistics aspects are also discussed. A further study of the course syllabus shows that aspects of society normally include topics of cultural norms, expectations, social contexts in which a language is used, and society’s effect on language. In comparison, less language courses focus on the intercultural components, and even fewer are about multi-cultural communication. Only one result was obtained from the keyword “multi culture,” indicating that very few language learning courses involve multi-cultural communication. We feel that this is because most language courses in MOOCs teach only one foreign language. It is assumed that communication subjects rather than foreign language learning subjects would focus more on multi-cultural components.

The findings of our study indicate a significant gap in the current state of language education courses in MOOCs. Although it is encouraging that more than 70% of courses have incorporated cultural components, the low percentage of cross-cultural, intercultural, or multicultural components is alarming. The development of intercultural competence is crucial for effective communication in today’s globalized world. As Kramsch (1993) argues that “language teaching is culture teaching” (p. 19), language learners need to understand the intercultural contexts in which the language is used. Language education courses in MOOCs have the potential to provide learners with a platform to acquire such skills. It is suggested that MOOCs need to prioritize the integration of intercultural components in their language education courses to better prepare learners for the complexities of intercultural communication.

• RQ2: Which languages and offering countries are involved in the MOOCs that promote intercultural competence?

Table 2 shows the numbers of MOOCs categorized by language and country. A total number of 13 languages were identified among the 138 MOOCs. The third column shows the number of MOOCs related to each language. The fourth column lists the number of country-wise distribution of MOOCs. As can be seen in Table 2, MOOCs offer courses to teach seven European languages, two aboriginal languages from Australia and New Zealand, and four East Asian languages, respectively. In terms of the number of courses, an overwhelming MOOCs (63.8%) teach Western languages or languages under Western influence, while only 36.2% teach East Asian languages including Chinese, the most influential one. Surprisingly, despite a significant number of their native speakers, Indian, Arabic, and native African languages are not included in the MOOCs surveyed. Hindi, Bengali, and Marathi languages, spoken by a population of 709 million people in India (Pathak, 2022), Arabic, spoken by 420 million people worldwide (Industry Arabic, 2023), and native African languages, spoken by more than 300 million people (Tirosh, 2021), were not represented. It is also noteworthy that all MOOC providers except Argentina are western or East Asian countries, further highlighting the lack of representation from other regions. The findings confirm the literature that MOOCs perpetuate a form of digital neocolonialism and educational hegemony, as Western languages and cultures continue to dominate the global education landscape (Altbach, 2014; Adam, 2019). Although the rise of Chinese becomes another influential force (Shadiev and Sintawati, 2020), the study reveals an evident bias towards Western languages and those that have been influenced by Western culture in MOOCs.

**Table 2:** Language-wise and country-wise distribution of MOOCs of language learning demonstrating intercultural competence.

No	Language	No.	Country wise distribution
1	English	36	United States (14), China (17), Spain (2), United Kingdom (3)
2	Chinese	34	China (24), United States (7), Australia (3)
3	Spanish	21	United States (7), Colombia (6), United Kingdom (4), Spain (3), Argentina (1)
4	Irish	13	Ireland (13)
5	Korean	8	Korea (7), Malaysia (1)
6	Italian	6	United States (4), Italy (2)
7	Japanese	6	Australia (3), China (2), Japan (1)
8	French	5	United Kingdom (3), Australia (1), France (1)
9	Norwegian	3	Norway (3)
10	Thai	2	Malaysia (2)
11	Portuguese	2	United Kingdom (1), Malaysia (1)
12	Noongar	1	Australia (1)
13	Te Reo Maori	1	New Zealand (1)

It is interesting to see that many languages courses are taught by a host country that does not use that language as the native language. For example, besides the native speaking country China, Chinese language is also taught by the host countries of United States and Australia. Japanese language is also taught by Australia and China. Further analysis of course content indicates that when a language course involves non-native speaking teacher and/or students, intercultural components are more likely to be embedded into the lectures. Here we use the course “Japanese Culture and Language I and II” offered by TsinghuaX in China as an illustration. Tsinghua University, founded in 1911, is one of the most prestigious universities in Asia. As China’s leading institution of advanced learning, Tsinghua is dedicated to excellence in education, research, and social services. It is one of the main MOOCs provider from China, which offers more than 70 courses in edX and other platforms. The teachers of this course are all native Chinese professors/lecturers. The medium of instruction is Chinese. The target learners are Chinese students who learn Japanese as a foreign language. Table 3 demonstrates the excerpt of a lecture transcript, showing how intercultural components are embedded in the course.

**Table 3:** Excerpt of a lecture transcript of a Japanese course provided by TsinghuaX in China.

Transcript summary	Translation
日文的君字是一個來源於中文的漢字，若將此字念成「くん」，主要是用在對男生的稱呼，尤其是稱呼男同學或男性同輩、晚輩時經常使用。 另外一個念法為「きみ」，通常是年長紳士或男性長輩對男女晚輩的一種稱呼。 但若男生之間彼此立場相同時使用「君」的話，一種是表示親密，另一種則是指責或批評。 君字在中文的使用有非常不同的含義。第一種含義是封建時代指帝王、諸侯等, 如君主。君子。第二種含義是指品行好的人，如正人君子。第三種含義是對對方的尊稱, 例如張君，諸君。	The Japanese kanji *Jun* is originated from a Chinese character. If it is pronounced as “くん,” it is mainly used to address boys, especially when addressing male classmates or male peers or juniors. Another way to pronounce it is “きみ,” which is usually an address for male and female juniors by older gentlemen or male elders. However, if boys use “*Jun*” when they have the same position, one is to express intimacy, and the other is to accuse or criticize. In contrast, the character *Jun* is used in Chinese to have very different meanings. The first meaning refers to emperors or princes in the feudal era: such as monarch or gentleman. The second meaning refers to a person of good conduct, such as “a gentleman.” The third meaning is the respectful title for the other party such as in: Sir Zhang, Gentlemen.

Coming from the same cultural background as the students—Chinese culture, the teachers might be more aware of the language learning difficulties caused by cultural differences between China and Japan. In order to facilitate Chinese students to distinguish the use of *jun* in Japanese from that in Chinese, the teachers explained the cultural differences between China and Japan. Japanese Kanji *jun* is used between peers or to a younger generation, so the system of honorific speech is not required. In contrast, the Chinese character *jun* is used to show respect to people from higher hierarchy, such as an emperor in the ancient time. While the use of this word *jun* (Japanese kanji or Chinese character) is different, both Japan and China are hierarchical societies with high power distance (Hofstede, 1980). People should be conscious of their hierarchical positions in social settings and act accordingly. The comparison between different cultures is an important part of the learning of the Japanese Kanji *jun*. This example demonstrates that cultural factors are an important part of foreign language learning which cannot be ignored.

**Figure 3:**
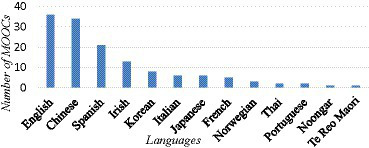
Languages involved in language learning MOOC offerings and their total count.

Figure 3 shows the languages taught in these language learning MOOCs and the number of MOOCs for each language. English, Chinese and Spanish are the top three languages taught in the MOOCs with IC components. This result is in line with a survey about most spoken languages (Lingua Language Center, 2022), which shows that English (1,121 million speakers), Chinese (1,107 million), Hindi (698 million) and Spanish (512 million) are most spoken in the world in 2022. Also, according to Shadiev and Sintawati’s (2020) review article on intercultural learning, English, Chinese and Spanish were among the four most frequently used languages in the reviewed articles. The finding also echoes Adam’s (2019) survey of around 9,600 MOOCs which found that more than half were produced in English, Mandarin Chinese and Spanish. It is believed that English, Chinese and Spanish courses are most offered in MOOCs because these languages are dominant languages for global business and academic communication. In terms of plurality of knowledge, it seems that MOOCs are promoting these dominant language and cultures while inhibiting others (Adam, 2019). Interestingly, Hindi, the third most spoken language in the survey (Lingua Language Center, 2022), was not even ranked top 10 in MOOCs. When we used “Hindi” as a keyword to search in edX, only one result came back (February 25, 2023)—“*Nitrogen: A Global Challenge (Hindi)*.” In this course, Hindi is used as a medium of instruction, not the language to be taught. Although efforts to promote Hindi overseas have been ongoing for decades, Hindi is not widely spoken outside India due to various historical reasons (Guha, 2020), and therefore, catches less attention in language education. Another possible reason is the influence of English language, which is widely used in India as a major official language along with Hindi.

**Figure 4:**
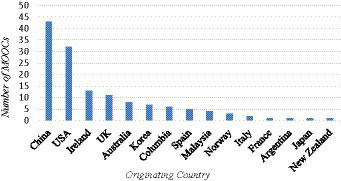
Originating countries of MOOCs of language learning demonstrating intercultural competency.

Figure 4 shows the number of language learning MOOCs offered by countries. China (43), United States (25), and Ireland (14) are the top three countries which offer the highest numbers of language teaching MOOCs with IC components, followed by the United Kingdom (11) and Australia (8). Our results are in line with those obtained in previous intercultural studies. For example, Shadiev and Sintawati’s (2020) research confirms that United States and China were leading in intercultural learning projects. Çiftçi (2016) and Avgousti (2018) also found that between 2004 and 2015 (February), the United States was the most frequently involved in intercultural learning projects. Our search result seems to indicate a connection between language education, intercultural research and publications. Among the top five countries that offer the highest number of language learning MOOCs, four are Western countries. The finding confirms, to some extent, the dominance of Western-centric epistemologies in MOOCs (Altbach, 2014; Adam, 2019). Our research resonates the previous studies that China is becoming another influential force (Shadiev and Sintawati, 2020). Although the MOOC platforms originate from the United States, China has become the country which offers the highest number of language learning MOOCs. This indicates that, when language learning MOOCs are concerned, Western-centric knowledge is not the only dominant force. With China being one of the largest economies in the world, the elite universities in China have resources to support the design and offering of MOOCs. While serving the branding function for the universities, these MOOCs also promote the Chinese language and culture to global audiences.

**Figure 5:**
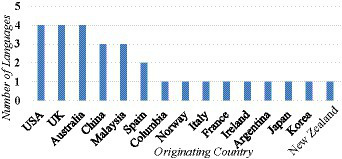
Originating countries of MOOCs of language learning and number of languages involved.

Figure 5 shows that regarding the diversification in languages, United States, United Kingdom and Australia offer the higher number of language learning MOOCs on different languages. As shown in Table 2, the language learning MOOCs offered by the United States teach English, Chinese, Spanish, and Italian. Those offered by the United Kingdom teach English, Spanish, French, and Portuguese. Those offered by Australia teach Chinese, Japanese and Noongar. This also indicates that, in most of the English-speaking countries, there is a growing interest in learning or teaching different languages which are not native to them. In addition to the dominant foreign languages such as Chinese or Spanish, learners are given more choices to learn a foreign language they like.

To sum up, the findings of this study suggest that population size may not be a significant predictor of the selection of languages taught in MOOCs. This study reveals the existence of a biased attitude towards Western languages and the ones influenced by Western culture, with Asian languages start to receive some attention. Despite some progress towards diversification, this study emphasizes the need for a plurality of languages and cultures to be taught through MOOCs. Moreover, it is important to consider the epistemological framework underpinning the MOOCs. Although China is becoming another influential force (Shadiev and Sintawati, 2020), if the courses reflect dominant Western-centric, learners, particularly those from regions not covered in this study, may struggle to think critically about different cultures and traditions. Therefore, a more inclusive and diverse approach to language education in MOOCs is necessary to promote intercultural communication and understanding (Altbach, 2014; Adam, 2019).

• RQ3: What soft skills are embedded in the language learning courses with IC components in MOOCs?

We have gathered the soft skill areas fostered by these language learning MOOCs which are responsible for promoting IC development among learners (Table 4). After analyzing all the language learning MOOC titles and their brief course introduction, we have found that language learning MOOCs contribute to five types of soft skills. These skills are (a) language skills, (b) communication skills, (c) business and entrepreneurship skills, (d) career development skills, and (e) cultural development skills. The respective skill areas are listed in Table 4. Interestingly, in addition to business and entrepreneurship skills, the language learning MOOCs also promote career development skills such as journalism, media literacy, travelling, instruction for academics, science, technology, engineering, mathematics, and sports. Moreover, they also prepare learners for language tests such as HSK (Hànyŭ Shuiˇpíng Kǎoshì, a test of Chinese language proficiency), and the study of AP (Advanced Placement) courses of United States by providing an opportunity for learners to earn college credit in high school.

**Table 4:** Intercultural competence related soft skills and skill areas which are fostered by language learning MOOCs.

Soft Skills	Skill areas
Language	reading, writing, vocabulary, writing professional E-mails, language skills for non-native speakers, language in society, grammar, time and direct object pronouns, prepositions, adverbs, and tenses
Communication	communication skills, speaking English professionally: in person, online and on the phone, communication in healthcare, pronunciation for communication, conversation skills, speeches, presentations, networking in English, writing, presenting and submitting scientific papers in English, global communication, people and their interests, meeting people and describing places
Business and entrepreneurship	business English, finance and economics, management and leadership, business speaking, business writing, business and globalization, business and technology
Career development	journalism, media literacy, travelling, instruction for academics, science, technology, engineering, and mathematics, academic, HSK Test (Hànyuˇ Shuiˇpíng Kǎoshì), AP^®^ (Advanced Placement) English literature (stories, poems, and plays), sports
Cultural development	cultural experience, cross-cultural communication, heritage and popular culture, intercultural communication, literature, cinema and the visual arts, traditions and celebrations, social culture, contemporary culture, cultural connections, culture and language, cultural information for travelers, home, people and places

Today, we live in a digital world without any barriers or boundaries which greatly facilitates international communication, so understanding cross-cultural and intercultural aspects plays a major role especially for those in leadership and managerial positions. As many of us are deeply interconnected today either through the Internet or virtual world, most of our daily activities involve deep interactions with virtual platforms. MOOCs provide a new dimension to online learning, and it is easy for anyone to access course contents through the Internet. With thousands of MOOCs, there are plenty of opportunities for learning all kinds of skills including soft skills and hard skills. Communication, teamwork, creativity, and problem solving are examples of soft skills. Skills on computer programming, science, and engineering are examples of hard skills. In Table 4, while most of the skills are soft skills, there are some hard skills such as media literacy, engineering, and mathematics, etc. These hard skills are listed in the table because they are fostered in language courses such as “English for Science, Technology, Engineering, and Mathematics.” This shows that some MOOCs help to improve effective communication in diversified disciplines. Similarly, the language courses help us to enhance various other soft skills such as collaboration, team management, negotiation skills, adaptability, active listening, public speaking, friendliness, ethics and inclusiveness, etc.

## 5. Conclusion

To conclude, this study has identified culture-related keywords embedded in the titles and introduction of language learning MOOCs (RQ1). After repeated searching and screening, this study identifies five keywords related to intercultural competence, rather than using the keywords “language” or “communication.” We then reduced the redundancy and obtained 138 unique MOOCs on 13 different languages offered by 15 countries through 42 universities/organizations. We find that the majority of language education courses in MOOCs are categorized under the keyword “culture,” which aligns with the literature that emphasizes the inseparability of language and culture (Byram, 1997). Language learning is not just the mastery of words, grammatical principles, and sentence construction, but also its unique cultural norms, social systems, and cognitive processes. Understanding these cultural-specific contexts along with linguistic principles is central to effective language acquisition. It is concerning that only less than a quarter of MOOC language courses include cross-cultural, intercultural, or multicultural components since intercultural competence is crucial for effective communication in the current globalized world. Therefore, MOOCs should prioritize integrating intercultural components into their language education courses to assist learners in preparing for intercultural communication.

Second, regarding what countries offer what languages in MOOCs promoting intercultural competence (RQ2), it was found that English, Chinese and Spanish are three most learned languages. This result is in line with the languages most spoken across the world. China, United States, Ireland, United Kingdom and Australia are the five countries that promote most language courses with IC components. United States, United Kingdom and Australia each offers language learning MOOCs on four different languages, providing the most diversified language learning choices. The results indicate that a limited number of languages and offering countries dominate the language learning MOOCs. While previous studies have identified the dominance of the Western-centric epistemologies in MOOCs, this study identifies both Western countries and China as major influencing forces in language learning MOOCs with IC components. It is suggested that more acknowledgment be given to the marginalized knowledge cultures and more opportunities to diversified languages and cultures on MOOCs. MOOCs developers can avoid further reinforcing dominant epistemologies while ignoring the marginalized ones, resulting in more epistemic injustices. In this way, MOOC platforms can truly embrace inclusion and plurality.

This study also finds that these language courses in MOOCs cultivate five types of soft skills (language learning skills, communication skills, business and entrepreneurship skills, career development skills, and cultural development skills; RQ3). MOOCs have demonstrated the potential to cultivate learners who not only possess the ability to speak a foreign language, but also can acquire additional soft skills required for people to be effective communicators in professional settings.

This study shows that embedding IC skills in language learning MOOCs is a common trend. MOOCs play a very important role in remote learning, as well as in more desirable situations such as a global pandemic. In this study, the language learning MOOCs fostering soft skill development available from four MOOC providers are extensively surveyed (Coursera, edX, FutureLearn, and Open Universities Australia, as of October 18, 2022).

Despite these insightful findings, some limitations must be acknowledged. As the study only focuses on MOOCs and lists the courses which reflect cultural competence in terms of course contents, there is less emphasis on how learners can enhance cultural competence by taking these courses. This study does not collect data on how students really interact with each other to improve their intercultural competences and soft skills, for example learning a foreign language through either interacting with fellow learners or with instructors. As the learning happens remotely, it is not easy to gather the actual information on learners’ progress in developing intercultural competence skills. It is recommended that further research should collect empirical data to investigate how teacher-student interaction can influence learners’ acquisition of intercultural competences and soft skills in language education classes. Secondly, there is not much information on students’ background, and their native language, and reasons for joining these courses. However, it is evident from course titles that there are several MOOCs which are available for global learners to improve their skills in speaking, reading, writing, pronunciation and e-mail communication. Some of the MOOCs also provide skills on negotiation, cross-cultural communication, and networking. Another limitation of this study is its sole focus on language learning MOOCs. MOOCs which focus on other aspects of developing intercultural competence are not examined. As the initial search results are in huge numbers, it is not possible to analyze all the courses. The other limitation is that we do not adopt any established model in the analysis.

## Data availability statement

The original contributions presented in the study are included in the article/Supplementary material, further inquiries can be directed to the corresponding author.

## Author contributions

All authors listed have made a substantial, direct, and intellectual contribution to the work and approved it for publication.

## Funding

This work was supported by Natural Science Foundation of Shandong Province under Grant ZR2022MG015.

## Conflict of interest

The authors declare that the research was conducted in the absence of any commercial or financial relationships that could be construed as a potential conflict of interest.

## Publisher’s note

All claims expressed in this article are solely those of the authors and do not necessarily represent those of their affiliated organizations, or those of the publisher, the editors and the reviewers. Any product that may be evaluated in this article, or claim that may be made by its manufacturer, is not guaranteed or endorsed by the publisher.

## Footnotes

^1^ www.coursera.org

^2^ www.edx.org

^3^ www.futurelearn.com

^4^ www.udacity.com

^5^ https://novoed.com

^6^ https://iversity.org

^7^ www.canvas.net

^8^ www.open.edu.au

^9^ www.openlearning.com

^10^ www.udemy.com

^11^ https://alison.com
