# Supplementary material for: Massive Open Online Courses and intercultural competence: analysis of courses fostering soft skills through language learning
Source: Front Psychol. 2023 Aug 4;14:1219478. doi: 10.3389/fpsyg.2023.1219478 (PMC10436504; doi:10.3389/fpsyg.2023.1219478)
Supplement: Supplementary file 1 [file Data_Sheet_1.docx]

**Supplementary Materials**

**Title: MOOCs and Intercultural Competence: Analysis of Courses Fostering Soft Skills through Language Learning**

Laxmisha Rai^1^, Chunrao Deng^2^, Shuyang Lin^2^, Liu Fan^3*^

**APPENDIX A: MOOCs from Coursera**

| Codes:   - C: culture - CC: cross culture - IC: intercultural - MC: multi culture - S: society | | | |
| --- | --- | --- | --- |
| **No** | **Name of MOOCs** | **Language** | **Country** |
| **1** | English for Business and Entrepreneurship (C)  English for Media Literacy (C)  English for Science, Technology, Engineering, and Mathematics (C)  English for Career Development (C)  English for Journalism (CC) | English (5) | UPENN, USA |
| **2** | Learn Spanish: Basic Spanish Vocabulary (C)  Spanish Vocabulary: Cultural Experience (C)  Spanish Vocabulary: Sports, Travel, and the Home (C)  Spanish Vocabulary Project (C) | Spanish (4) | UCD, USA |
| **3** | Write Professional Emails in English (C, CC)  Take Your English Communication Skills to the Next Level (C, CC)  Speak English Professionally: In Person, Online & On the Phone (C) | English (3) | GIT, USA |
| **4** | Business English: Finance and Economics (C)  Business English: Management and Leadership (C) | English (2) | ASU, USA |
| **5** | Spanish for Successful Communication in Healthcare Settings (C) | Spanish (1) | RICE, USA |
| **6** | Étudier en France: French Intermediate course B1-B2 (C, IC) | French (1) | EP, France |
| **7** | Learn English (C)  Conversational English Skills (C)  Just Reading and Writing English 1 (C)  Just Reading and Writing English 2 (C)  General Academic English (C) | English (5) | TU, China |
| **8** | Chinese for Beginners (C)  More Chinese for Beginners (C)  Learn Chinese: HSK Test Preparation (C)  Chinese for HSK 6 (C)  Essentials for English Speeches and Presentations (C) | Chinese (4)  English (1) | PU, China |
| **9** | Business English: Basics (C, CC)  Business English for Non-Native Speakers (C, IC, CC)  Business English for Cross-cultural Communication (C, IC, CC)  English for Effective Business Speaking (C)  English for Effective Business Writing (C, CC) | English (5) | HKUST, China |
| **10** | Mandarin Chinese 1: Chinese for Beginners (C)  Mandarin Chinese 2: Chinese for Beginners (C)  Mandarin Chinese 3: Chinese for Beginners (C)  Mandarin Chinese for Intermediate Learners: Part 1 (C)  Mandarin Chinese for Intermediate Learners: Part 2 (C)  Mandarin Chinese for Intermediate Learners: Part 3 (C)  Mandarin Chinese for Intermediate Learners: Capstone Project (C) | Chinese (7) | SJTU, China |
| **11** | A Bridge to the World: Korean Language for Beginners Ⅰ(C)  A Bridge to the World: Korean Language for Intermediate1 (C)  A Bridge to the World: Korean Language for Advanced Ⅰ(C) | Korean (3) | SU, Korea |
| **12** | First Step Korean (C) | Korean (1) | Yonsei, Korea |

**APPENDIX B: MOOCs from edX**

| **No** | **Name of MOOCs** | **Language** | **Country** |
| --- | --- | --- | --- |
| **1** | Chinese Language in Culture: Level 1 (C, IC, S)  Chinese Language in Culture: Level 2 (C, IC, S) | Chinese (2) | MIT, USA |
| **2** | AP® English Literature & Composition – Part 1: Stories (C, IC)  AP® English Literature & Composition – Part 2: Poems (C, IC)  AP® English Literature & Composition – Part 3: Plays (C, IC) | English (3) | UCB, USA |
| **3** | Preparing to Network in English (C) | English (1) | UW, USA |
| **4** | Italian Language and Culture: Beginner (2021-2022) (C)  Italian Language and Culture: Advanced (2021-2022) (C)  AP® Italian Language and Culture (2022-2023) (C, S)  Italian Language and Culture: Intermediate (2021-2022) (S) | Italian (4) | WC, USA |
| **5** | AP® Spanish Language and Culture (C) | Spanish (1) | BU, USA |
| **6** | Noongar Language and Culture (C, IC, S) | Noongar (1) | CU, Australia |
| **7** | AP® Spanish Language and Culture (C, IC, CC) | Spanish (1) | SMES, USA |
| **8** | A travel by Spanish America: Spanish for beginners (C) | Spanish (1) | UR, Columbia |
| **9** | Spanish Across the Americas (C, IC, CC) | Spanish (1) | UNC, Argentina |
| **10** | Spanish for beginners: Learn in paradise (C) | Spanish (1) | UJ, Columbia |
| **11** | Italian Language around the world (C) | Italian (1) | USNF, Italy |
| **12** | Upper-Intermediate English: Business and Globalization (C)  Basic Spanish 1: Getting Started (C, IC)  Basic Spanish 2: One Step Further (C, IC)  Basic Spanish 3: Getting there (C, IC)  Upper-Intermediate English: Business and Technology (C, IC) | Spanish (3)  English (2) | UPV, Spain |
| **13** | Japanese Culture and Language (I) (C, IC)  Japanese Culture and Language (II) (C, IC)  Just Reading and Writing in English (C)  Conversational English Skills (C, IC)  Writing, Presenting and Submitting Scientific Papers in English (C, IC)  General Academic English (S) | Japanese (2)  English (4) | TU, China |
| **14** | English for Doing Business in Asia – Speaking (C, IC, CC)  English for Doing Business in Asia – Writing (C, IC, CC) | English (2) | HKUST, China |
| **15** | Mandarin Chinese for Business (C)  Mandarin Chinese Essentials (C)  Mandarin Chinese Level 1 (C)  Mandarin Chinese Level 2 (C)  Mandarin Chinese Level 3 (C) | Chinese (5) | MandarinX, USA |
| **16** | Japanese Pronunciation for Communication (C, S) | Japanese (1) | Waseda, Japan |

**APPENDIX C: MOOCs from FutureLearn**

| **No** | **Name of MOOCs** | **Language** | **Country** |
| --- | --- | --- | --- |
| **1** | Irish 101: An Introduction to Irish Language and Culture (C)  Irish 102: An Introduction to Irish Language and Culture (C)  Irish 103: An Introduction to Irish Language and Culture (C, S)  Irish 104: An Introduction to Irish Language and Culture (C, S)  Irish 105: An Introduction to Irish Language and Culture (C)  Irish 106: An Introduction to Irish Language and Culture (C)  Irish 107: An Introduction to Irish Language and Culture (C)  Irish 108: An Introduction to Irish Language and Culture (C)  Irish 201: An Introduction to Irish Language and Culture (C)  Irish 202: Irish Language and Culture for Adults (C)  Irish 203: Irish Language and Culture for Adults (C)  Irish 204: Irish Language and Culture for Adults (C)  Irish 205: Irish language and Culture for Adults (C) | Irish (13) | DCU, Ireland |
| **2** | Intermediate Spanish: The Worlds of Spanish (C)  Intermediate Spanish: Leisure in Spanish-Speaking Countries (C, S)  Intermediate Spanish: Literature, Cinema and the Visual Arts (C, S)  Intermediate Spanish: Heritage and Popular Culture (S)  Intermediate French: Discovering Worlds of French (C)  Intermediate French: Traditions and Celebrations (C)  Intermediate French: People and Places (IC) | Spanish (4)  French (3) | OU, UK |
| **3** | English Grammar: All You Need to Know (C) | English (1) | UCL, UK |
| **4** | Foundations of Portuguese for Global Communication (C) | Portuguese (1) | KCL, UK |
| **5** | Exploring English: Language and Culture (C) | English (1) | BC, UK |
| **6** | Learn Spanish 1: Basics, meeting people and describing places (C)  Learn Spanish 2: People and their interests (C)  Learn Spanish 3: Time and Direct Object Pronouns (C)  Learn Spanish 4: Spanish Prepositions, Adverbs, and Tenses (C) | Spanish (4) | UPB, Colombia |
| **7** | Introduction to Norwegian (C, S) | Norwegian (1) | UO, Norway |
| **8** | Norwegian for Beginners 2 (C)  Norwegian for Beginners 3 (C) | Norwegian (2) | NUST, Norway |
| **9** | Introduction to Italian (C) | Italian (1) | USS, Italy |
| **10** | Introduction to Korean (C)  Introduction to Korean: 2 (C)  Intermediate Korean: Exploring the Language and Culture of Korea (C) | Korean (3) | HU, Korea |
| **11** | Let's Learn Thai Language (C)  Let’s Learn Thai Language: Basic Conversation Skills (C)  Fun in Korea: Useful Expressions and Cultural Information for Travellers (C)  Let's Learn Portuguese Language (C) | Thai (2)  Korean (1)  Portuguese (1) | UM, Malaysia |
| **12** | Te Reo Māori: Introduction to Pronunciation (C, S) | Te Reo Māori (1) | EM,  New Zealand |
| **13** | HSK Standard Course Level 1: 1.1 (C)  HSK Standard Course Level 1: 1.2 (C)  HSK Standard Course Level 2: 2.2 (C)  HSK Standard Course Level 2: 2.3 (C)  Contemporary Chinese I: 1.1 (C, S)  Contemporary Chinese I: 1.2 (C, S)  Contemporary Chinese II: 2.1 (C)  Contemporary Chinese II: 2.2 (C)  Contemporary Chinese III: 3.1 (C, MC)  Contemporary Chinese III: 3.2 (C, S)  Contemporary Chinese IV: 4.1 (C)  Contemporary Chinese IV: 4.2 (C) | Chinese(12) | CP, China |
| **14** | Learn Chinese: Introduction to Chinese Conversation (C) | Chinese (1) | SISU, China |
| **15** | English as a Medium of Instruction for Academics (IC) | English (1) | US, UK |

**APPENDIX D: MOOCs from OU-A**

| **No** | **Name of MOOCs** | **Language** | **Country** |
| --- | --- | --- | --- |
| **1** | Social Culture in Chinese Reading I (C)  Social Culture in Chinese Reading II (C)  French Through Contemporary Culture (C)  Introduction to Japanese Language in Society (S) | Chinese (2)  French (1)  Japanese (1) | UNE, Australia |
| **2** | Advanced Chinese: Cultural Connections (C)  Advanced Japanese: Cultural Connections (C)  Advanced Japanese: Intercultural Communication (IC, MC) | Chinese (1)  Japanese (2) | CU, Australia |
